# Characterization of host immune cell infiltrate in human CAR T cell-mediated xenogeneic graft versus host disease in NSG mice

**DOI:** 10.1177/03009858251391388

**Published:** 2025-11-19

**Authors:** Elinor Willis, Esha Banerjee, Jillian Verrelle, Arin Cox, Charles-Antoine Assenmacher, Enrico Radaelli

**Affiliations:** 1 Penn Vet Comparative Pathology Core, Department of Pathobiology, School of Veterinary Medicine, University of Pennsylvania, Philadelphia, PA; 2 Altasciences Preclinical, Seattle, WA

**Keywords:** CAR T cell, graft versus host disease, immunohistochemistry, macrophage polarization, NSG mice, xenogeneic

## Abstract

Chimeric antigen receptor (CAR) T cells are revolutionary cancer therapies that are Food and Drug Administration-approved for hematologic malignancies and under investigation for solid tumors. The use of allogeneic over autologous CAR T cells offers advantages, including broader availability and reduced costs. However, allogeneic CAR T cells frequently trigger graft versus host disease (GvHD), a complication observed in patients and experimental models where human CAR T cells are delivered into immunocompromised mice. To understand the contribution of the mouse immune response to human CAR T cell-mediated xenogeneic GvHD, we analyzed GvHD lesions in a human xenograft tumor model in NOD.Cg-*Prkdc*^scid^ *Il2rg*^tm1Wjl^/SzJ (NSG) mice. The animals were treated with second-generation CAR T cells targeting a human tumor-specific antigen without a murine homolog. Mice treated with CAR T cells had more severe GvHD lesions than control mice receiving nontransduced (NT) T cells. Also, tumor burden was negatively correlated with GvHD lesion severity. Immunohistochemical characterization of the GvHD lesions showed that approximately 45% of the immune cell infiltrate consisted of murine cells, most of which were IBA1+ histiocytes, with a small population of CD11c+ dendritic cells. The murine histiocytes expressed activation/antigen presentation markers, including high levels of the costimulatory molecule CD86. Analysis of macrophage polarization indicated an M2-like phenotype. These findings demonstrate a significant contribution of the mouse histiocytic compartment to lesions of human CAR T cell-mediated xenogeneic GvHD. Our results suggest that CD86+ murine antigen-presenting cells help trigger and sustain the xenoreactive CAR T cell response. Furthermore, xenogeneic GvHD exhibits a shift toward M2 polarization in murine macrophages.

Chimeric antigen receptor (CAR) T cells are groundbreaking therapies for hematologic malignancies and are under intense investigation for solid tumors as well as expanded indications, such as autoimmune diseases.^18,37^ Current CAR T cell therapies are derived from the patient’s own (autologous) T cells. This approach is limited by long manufacturing times, cost, and possible patients’ T cell dysfunction due to immune dysregulation and/or prior treatment(s).^6^ Therefore, intense research efforts are currently devoted to the development of “off-the-shelf” allogeneic CAR T cells, which could be produced in large batches from healthy donors and stored for immediate use, avoiding treatment delays, high cost, and impaired T cell activity.^6,36^ However, while promising in many respects, allogeneic CAR T cells carry an increased risk of graft versus host disease (GvHD), as demonstrated in clinical trials of pediatric and adult patients.^3,30,36^

The main preclinical platform for CAR T cell studies is xenogeneic delivery of human CAR T cells in immunocompromised mice.^8,40,44^ Also in this purely experimental setting, xenogeneic GvHD (xGvHD) emerges as one of the main complications associated with human CAR T cell administration.^8^ The progressive clinical evolution of this disorder leads to premature mortality among treated animals, limiting the assessment of long-term study endpoints important for efficacy evaluation, such as CAR T cell persistence within the host and tumor immune surveillance ability.^8,27^ In addition, the clinicopathological changes caused by xGvHD may represent a confounding factor for the interpretation of both anti-tumor efficacy and potential adverse events, such as on-target/off-tumor effects, caused by CAR T cell treatment.^8^

xGvHD has been extensively described and characterized, especially in immunocompromised mice engrafted with human peripheral blood mononuclear cells.^11^ Examples of xGvHD have also been reported in other experimental settings, including patient-derived tumor xenograft models and mice humanized with CD34+ hematopoietic stem cells.^28,32,39,43^ However, the pathogenesis of this condition has not been completely elucidated yet.^9,10^ In general terms, xGvHD appears to be driven by a coordinated cell-mediated immune response that is set in motion by xenoreactive human T cells recognizing the recipient mouse tissues as non-self.^9,17^ The primary predisposing factor is the highly permissive environment of the severely immunocompromised murine recipients, such as NOD.Cg-*Prkdc*^scid^ *Il2rg*^tm1Wjl^/SzJ (NSG) mice, lacking critical components of both the innate and adaptive immune response.^9,10,17^ Based on a series of studies aimed at elucidating the key triggering factors of xGvHD, the xenoreactive response of human CD8 cytotoxic T cells emerged as the main initiating mechanism.^41^ This happens upon priming of human CD8 T cells against mouse xenoantigens via the interaction between the MHC class I molecule on mouse antigen-presenting cells (APCs) and the T cell receptor on human T cells.^20^ In the context of this interspecies immune synapse, the positive costimulatory signal mediated by human CD28 binding with mouse CD86 is also critical to promote an effective cytotoxic response.^41^ The immune interface between human CD4 T cells and mouse APCs expressing MHC class II plays an important role in amplifying the xenoreactive response initiated by human CD8 T cells, but it is not, per se, sufficient to initiate xGVHD.^10,20^

Despite the drastic increase in the risk of developing xGvHD in the context of human CAR T cell delivery in immunocompromised mice, studies specifically addressing and investigating the pathogenesis of this disorder in preclinical models of cellular immunotherapy are limited.^13^ It is assumed that the xenogeneic immune attack perpetrated by human CAR T cells shares similar pathological features with xGvHD observed in other experimental contexts. However, little is known about the contribution of the mouse host response in determining the nature and severity of xGvHD in different organs and tissues. The exact composition of host inflammatory/immune cell phenotypes within these lesions has not been previously investigated. In that regard, this study aims to characterize the host immune cell populations coexisting with xenoreactive human T cells and their association with lesion severity in the setting of xGvHD induced by human CAR T cells in NSG mice.

## Materials and Methods

### Animals

Three and a half-month-old NSG mice received a single dose of 1.5 × 10^6^ proprietary human CAR T cells intravenously via tail vein injection. All mice were male as the carcinoma under investigation more commonly affects males. The CAR T cells were second generation (ie, one costimulatory domain fused to the signaling domain)^18^ and targeted against a human tumor-specific antigen without a murine homolog. Eight different CAR constructs were used in the study. Each construct contained one of two costimulatory domains (designated A and B) with one of four additional modifications to the costimulatory domain. Nine groups of mice were included in the study, with 5 mice per group. Eight groups received CAR T cells transduced with one of the above-described constructs (ie, A1–A4 and B1–B4). The ninth group received NT human T cells. Seven days before CAR T cell administration, all mice received an intraperitoneal injection of a human carcinoma cell line that expressed the target recognized by the CAR T cells. All CAR T cells and NT T cells were obtained from the same healthy donor. The CAR T cell target, adopted cancer cell line, costimulatory domains, and all CAR constructs are considered confidential/proprietary information and therefore cannot be disclosed at this time. All animal experiments were performed following the Institutional Animal Care and Use Committee guidelines of the University of Pennsylvania (IACUC protocol no. 805789).

### Pathological Assessment

Mice were euthanized by carbon dioxide asphyxiation 6 weeks after CAR T cell administration and complete necropsies with macroscopic examinations were performed. A comprehensive panel of organs/tissues, including whole head (brain, eyes and adnexa [including Harderian glands], oral cavity, nasal passages, ears [including Zymbal’s glands], and pituitary gland), skin (dorsal region, auricle, and muzzle), salivary glands with cervical lymph nodes, larynx, trachea, esophagus, lungs, heart, liver, pancreas, kidneys, adrenal glands, gastrointestinal tract (stomach, duodenum, jejunum, ileum, cecum, proximal colon, and distal colon/rectum), gonads and reproductive tract, urinary tract, external genitalia, spleen, spine, sternum, mesentery with mesenteric lymph nodes, and quadriceps femoris, as well as gross lesions, were collected and fixed in 10% neutral-buffered formalin for histologic assessment. The head and sternum were decalcified in a 15% formic acid solution for 24 hours.

Formalin-fixed tissue samples were trimmed according to a standardized approach for rodent studies adapted from the Registry of Industrial Toxicology Animal Data (RITA) guidelines.^21,29,35^ Trimmed tissue samples were then routinely processed for paraffin embedding, sectioning, and hematoxylin and eosin staining. A full set of slides was evaluated for each animal. Lesions consistent with xGvHD were assigned a 0–4 semiquantitative score (minimal to severe) based on the amount of mononuclear cell infiltrate and extent and type of tissue damage as described in Supplemental Table S1. The degree of carcinomatosis was assessed microscopically by counting the number of neoplastic implants on the peritoneal surface of the abdominal organs, mesenteric ligaments, and other peritoneal membranes. Each neoplastic implant was categorized as small if completely contained within the 20× objective field of view (1.3 mm diameter, Olympus UPlanFL 20× objective), medium if exceeding the 20× objective field of view but completely contained within the 5× objective field of view (5.3 mm diameter, Olympus UPlanFL 5× objective), and large if larger than the 5× objective field of view. A carcinomatosis burden value was then calculated for each mouse, assigning a score of 0.25, 1, and 2 to each small, medium, and large implant, respectively.

### Immunohistochemistry

Immunohistochemistry (IHC) was employed as the main approach to study the nature and distribution of the mononuclear cell infiltrate associated with xGvHD lesions in the most consistently affected tissues. IHC was performed on animals selected from 3 different experimental groups (*n* = 4 per group): the most severely affected A4 group (CAR T cells with costimulatory domain A and additional modification 4), the B4 group (CAR T cells with costimulatory domain B and the matching additional modification 4), and the NT group. For IHC, 5 µm thick paraffin sections were mounted on ProbeOn slides (Thermo Fisher Scientific). The IHC procedure was performed using a Leica BOND RXm automated platform combined with the Bond Polymer Refine Detection kit (Leica no. DS9800). Briefly, after dewaxing and rehydration, sections were pretreated with the epitope retrieval BOND ER2 high pH buffer (Leica no. AR9640) for 20 minutes at 98°C. Endogenous peroxidase was inactivated with 3% hydrogen peroxide for 10 minutes at room temperature. Nonspecific tissue-antibody interactions were blocked with Leica PowerVision IHC/ISH Super Blocking solution (PV6122) for 30 minutes at room temperature. The same blocking solution also served as diluent for the primary antibodies. Details regarding primary antibodies and IHC detection technique are listed in Supplemental Table S2. Primary antibodies were incubated on the sections for 45 minutes at room temperature. A biotin-free polymeric IHC detection system consisting of horseradish peroxidase conjugated anti-rabbit immunoglobulin G (IgG) was then applied for 25 minutes at room temperature. Immunoreactivity was revealed with the diaminobenzidine chromogen reaction. Slides were counterstained in hematoxylin, dehydrated in an ethanol series, cleared in xylene, and permanently mounted with a resinous mounting medium (Thermo Scientific ClearVue coverslipper).

### Image Analysis

The Aperio Versa 200 scanner was used for image acquisition. The scanned images had a resolution of 0.273 μm/pixel. Images were analyzed with QuPath.^2^ Regions of cellular infiltrate totaling at least 1 × 10^5^ μm^2^ per tissue were manually annotated, and chromogen-positive pixels were counted using positive cell detection. Positive cells per annotated region were expressed as percentages. Human- and mouse-specific CD45 percentages were normalized to each other, totaling 100%. The remaining murine markers were normalized to mouse-specific CD45.

### Statistical Analysis

Statistical analyses were performed using GraphPad Prism 9.4.1. The comparison of xGvHD severity scores across multiple groups was calculated by Kruskal-Wallis test with Dunn’s multiple comparisons test. The comparison of xGvHD severity scores between 2 groups was calculated by Mann-Whitney test. Spearman’s correlation analysis was used to study the relationship between xGvHD severity scores and the different inflammatory/immune cell population ratios or peritoneal carcinomatosis burden. *P* < .05 was considered significant.

## Results

### Histopathology of xGvHD Lesions

**Figure 1.**
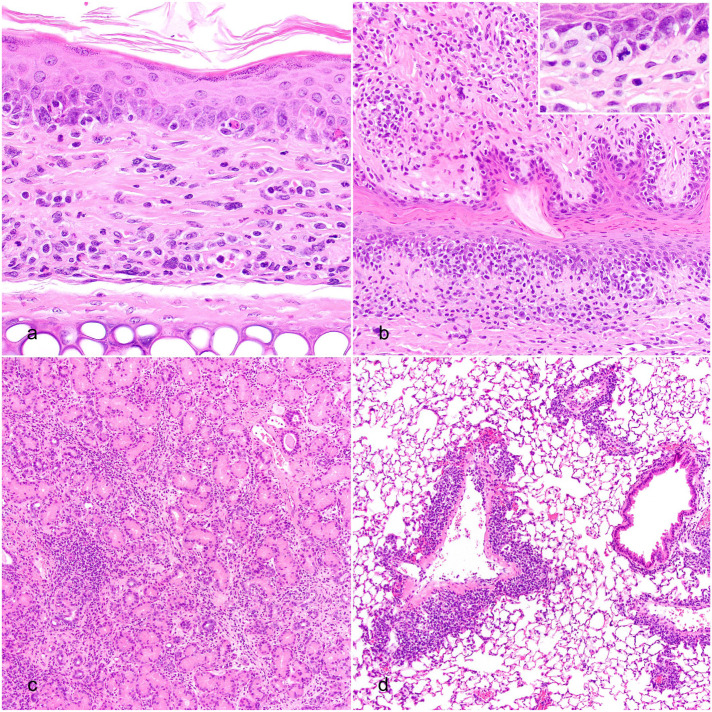
Histopathology of xenogeneic graft versus host disease lesions in mice treated with human chimeric antigen receptor (CAR) T cells. Hematoxylin and eosin. (a, b) Skin and mucous membranes exhibiting lichenoid interface dermatitis characterized by a mononuclear cell infiltrate targeting the dermoepidermal or mucosal-submucosal junction and epithelial cell death. Skin from the pinna (a) and mucosa of the penis (b). Inset in (b): high magnification of mitotically active and pleomorphic mononuclear cells. (c) Salivary gland with interstitial mononuclear cell infiltrates, fibrosis, and acinar loss. (d) Lung with perivascular and peribronchiolar mononuclear cell infiltrates.

All mice survived to the time of the scheduled necropsy at 6 weeks post-CAR T cell administration. xGvHD lesions consisted of infiltrates of mononuclear inflammatory/immune cells with evidence of host tissue damage, such as individual cell death, skin and mucosal membrane ulceration, parenchymal loss/atrophy, and, in severe cases, fibrosis (Fig. 1a–d). The most severely affected tissues included, in descending order, salivary and lacrimal glands, lungs, skin (especially of face and auricles), external genitalia, testis and epididymis, liver, Harderian and Zymbal’s glands, oral and nasal mucosae, and kidney. Other rarely affected tissues included accessory sex glands, esophagus, pancreas, stomach, and peripheral nervous system. The infiltrate of mononuclear inflammatory/immune cells affecting the skin (with adnexa) and mucosal surfaces exhibited a lichenoid interface pattern with basal epithelial cell degeneration/death and adnexal effacement/loss (Fig. 1a, b). In the skin, mast cells and granulocytes occasionally accompanied the mononuclear infiltrates. Lesions in glandular tissues were characterized by interstitial mononuclear cell infiltration, sometimes concentrated around ducts, with lobuloacinar atrophy/loss and fibrosis in the most severe cases (Fig. 1c). The lungs were affected by peribronchiolar, perivascular, and/or alveolar interstitial cellular infiltration with modest fibrosis and alveolar histiocytosis (Fig. 1d). Lymphoid tissues, in particular lymph nodes and spleen, were also highly infiltrated by mononuclear cells. In some tissues, the mononuclear cell population included large, mitotically active, mildly pleomorphic cells (Fig. 1b, inset).

### Severity of xGvHD Lesions Differs With Costimulatory Domains

**Figure 2.**
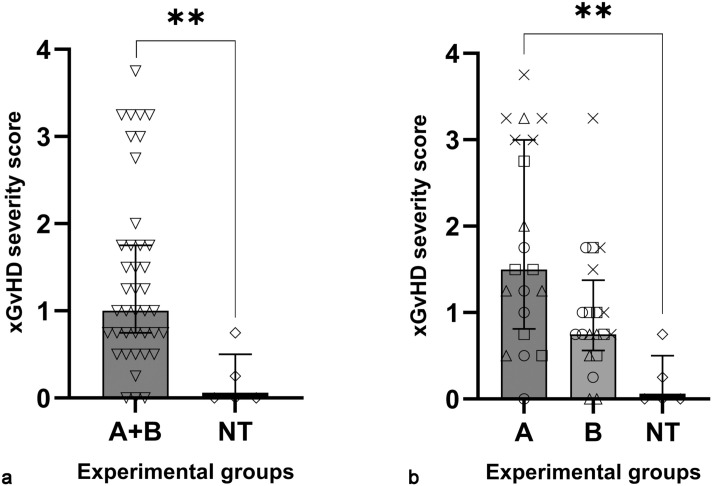
Severity of xenogeneic graft versus host disease (xGvHD) lesions differs by the presence and type of chimeric antigen receptor (CAR). (a) Mice that received nontransduced (NT) human T cells had less severe xGvHD lesions than mice that received CAR T cells (A+B). (b) Mice that received CAR T cells transduced with a construct containing costimulatory domain B had less severe xGvHD lesions than mice that received CAR T cells transduced with a construct containing costimulatory domain A. Each symbol (triangle, square, circle, and X) for A and B groups denotes one of the 4 additional modifications to the costimulatory domain. Mann-Whitney test (a) or Kruskal-Wallis test with Dunn’s multiple comparisons test (b), columns and error bars indicate medians and interquartile ranges, ** *P* < .01.

For each mouse, we computed a composite xGvHD severity score as the average of individual tissue xGvHD severity scores for the 4 most highly and consistently affected compartments (ie, salivary and lacrimal glands, lungs, skin, and external genitalia). Notably, the median composite severity score in the NT group was significantly lower (0 [interquartile range 0-0.5]) than the CAR groups (1 [interquartile range = 0.75–1.75]), reflecting milder xGvHD lesions or complete lack thereof (Fig. 2a). In addition, the overall severity of xGvHD lesions differed between groups based on the costimulatory domain and additional modification used in the CAR construct (Fig. 2b). The mice that received CAR T cells transduced with a construct containing costimulatory domain A had a higher median composite severity score than mice that received CAR T cells transduced with a construct containing costimulatory domain B (1.5 [interquartile range = 0.875–3] vs 0.75 [interquartile range 0.625–1.25], respectively). Despite these clear trends in severity, due to the relatively small sample size and high inter-individual variability within each group, only the comparison between groups A and NT reached statistical significance (Fig. 2b).

### Severity of xGvHD Lesions Is Negatively Correlated With Tumor Burden

All mice in the study received an intraperitoneal injection of a human carcinoma cell line before CAR T cell administration, enabling us to evaluate the relationship between anti-tumor efficacy and xGvHD lesions. In this context, statistical analysis indicated a strong negative correlation between the severity of xGvHD and the peritoneal tumor burden (Supplemental Figure S1).

### Immunohistochemical Characterization of Cellular Infiltrate Composition

To understand the composition of the cellular infiltrates, the mononuclear cells in xGvHD lesions in highly affected tissues (ie, salivary and lacrimal glands, lungs, skin, and external genitalia) were characterized by IHC. For this analysis, 3 groups were selected. First, we selected the group that had the highest xGvHD severity scores (group A4), which was one of the groups that received costimulatory domain A-containing CAR T cells. Second, we selected the group that received costimulatory domain B-containing CAR T cells with the matching additional modification (group B4). Mice that received NT T cells were also included in the analysis. IHC assays for diverse inflammatory/immune cell markers were applied to the xGvHD lesions, focusing on the characterization of the mouse component. The percentage of positive cells in each of the studied populations was calculated by digital image analysis.

**Figure 3.**
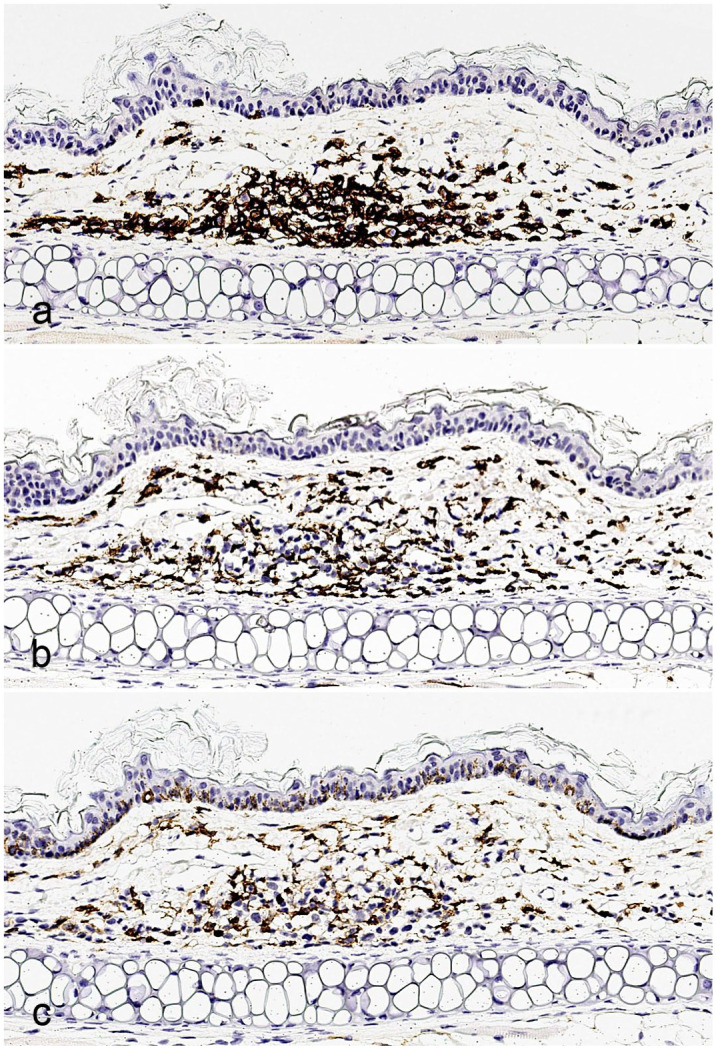
Xenogeneic graft versus host disease (xGvHD) lesions contain a substantial proportion of immune/inflammatory cells contributed by the mouse host. (a, b) Skin from the pinna with xGvHD lesion. Immunohistochemistry (IHC) for human-specific (a) and mouse-specific (b) CD45. (c) Skin from the pinna with xGvHD lesion. IHC for IBA1.

**Figure 4.**
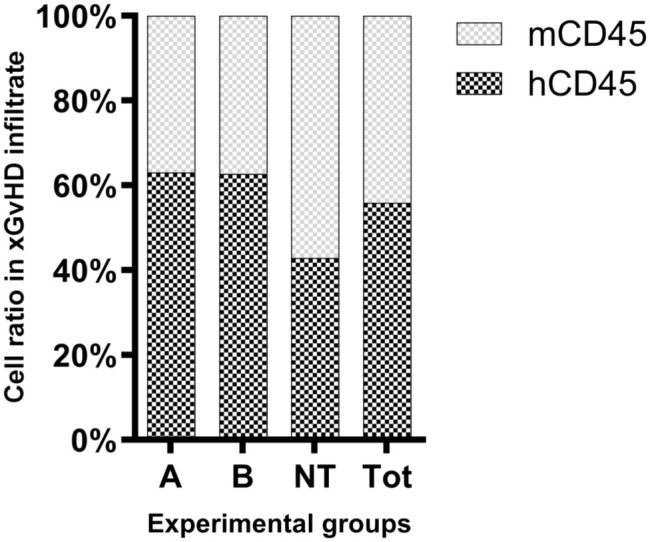
Ratio of cells positive by immunohistochemistry for human- and mouse-specific CD45 in the tissues most severely affected by xenogeneic graft versus host disease. Positive cells were quantified by digital image analysis. Data shown are medians within each experimental group (*n* = 4 mice/group). mCD45, mouse-specific CD45; hCD45, human-specific CD45; A, group treated with chimeric antigen receptor (CAR) T cells transduced with a construct containing costimulatory domain A; B, group treated with CAR T cells transduced with a construct containing costimulatory domain B; NT, nontransduced; and Tot, total.

We first determined the relative numbers of human and mouse inflammatory/immune cells in the xGvHD lesions by immunolabeling for matching broad-spectrum leukocyte markers: human- and mouse-specific CD45 (abbreviated as hCD45 and mCD45, respectively) (Fig. 3). Because of the experimental administration of purified human T cells (including both CAR and NT T cells), all hCD45+ cells were presumed to originate from the infused human T cell component. In addition, given the severely immunodeficient background of the treated NSG mice, with a complete lack of lymphoid cell expansion and maturation, all mCD45+ cells were interpreted as murine myeloid-derived immune/inflammatory cells. Overall, the mouse inflammatory/immune cell component accounted for over 40% of the infiltrate associated with xGvHD (Fig. 4). Across all mice with IHC data, a median of 56% (interquartile range = 53%–65%) of cells labeled positive for hCD45, while 44% (interquartile range 35%–47%) were positive for mCD45 (Fig. 4). Slight variations of hCD45 and mCD45 ratios were observed across treatment groups. However, the small sample sizes selected for the IHC study did not allow for any meaningful statistical comparisons. In addition, regardless of the treatment-group distributions, no statistically significant correlation could be traced between lesion severity scores and hCD45 or mCD45 ratios.

**Figure 5.**
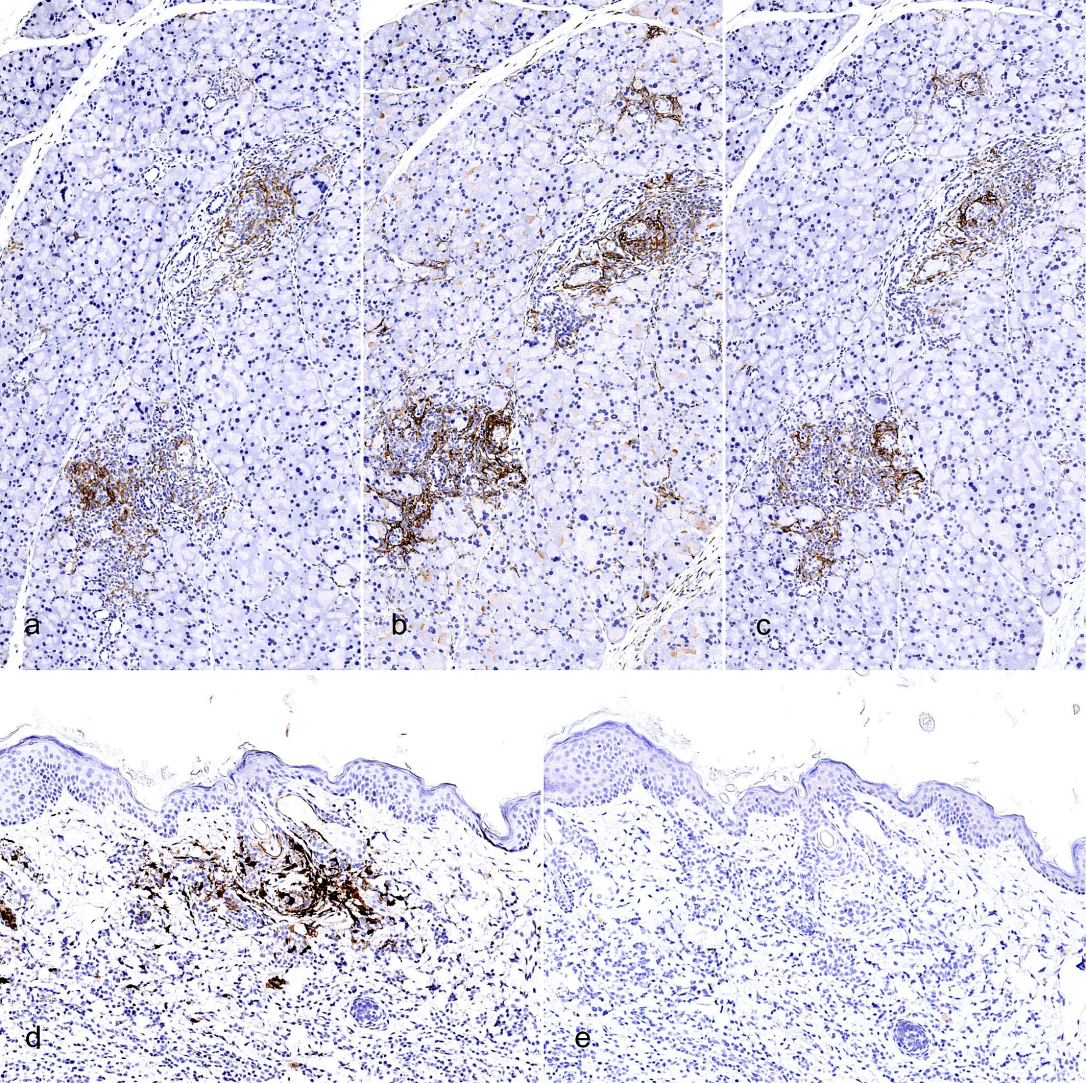
Characterization of activation and polarization status of histiocytes in xenogeneic graft versus host disease (xGvHD) lesions. (a, b, c) Salivary gland with xGvHD lesions. Infiltrating cells are positive by immunohistochemistry (IHC) for the dendritic cell marker CD11c (a), as well as for activation and antigen-presenting markers CD86 (b) and CD40 (c). (d, e) Haired skin with xGvHD lesions affecting adnexal epithelium. Infiltrating mouse histiocytes are strongly positive by IHC for the M2-like macrophage polarization marker arginase-1 (d) but negative for the M1-like macrophage polarization marker iNOS (e).

The mouse myeloid-derived component of the xGvHD infiltrate was further characterized by IHC using a battery of antibodies to better determine its phenotype and activation status. A median of 33% (interquartile range = 29%–40%) of all (human and mouse combined) infiltrating inflammatory/immune cells labeled positively for IBA1 (also known as AIF-1) (Fig. 3 and Supplemental Figure S2), demonstrating that the majority of mouse cells are histiocytic in origin, given that the human cells in the infiltrate exclusively included T cells, which do not express IBA1. Across examined xGvHD lesions, a relatively small but consistent fraction of these mouse histiocytes expressed the dendritic cell marker CD11c, accounting for a median of 7% (interquartile range = 6%–12%) of all (human and mouse combined) infiltrating inflammatory/immune cells (Fig. 5a and Supplemental Figure S2). To visualize the extent and activation status of mouse APCs within the xGvHD infiltrate, we performed IHC with mouse-specific antibodies targeting CD86 and CD40. Both of these markers were well-represented across all examined xGvHD lesions. However, mouse CD86+ APCs prevailed over mouse CD40+ APCs with a median of 18% CD86+ cells (interquartile range = 14%–23%) versus a median of 6% CD40+ cells (interquartile range = 3%–9%) across all (human and mouse combined) infiltrating inflammatory/immune cells (Fig. 5b, c and Supplemental Figure S2). While a certain degree of variability was observed for IBA1, CD11c, CD40, and CD86 across treatment groups, the small sample sizes selected for the IHC study did not allow for any meaningful statistical comparisons (Supplemental Figure S2). In addition, regardless of the treatment-group distributions, no statistically significant correlation could be traced between lesion severity scores and IBA1, CD11c, CD40, and CD86 ratios.

Finally, we sought to determine the mouse macrophage polarization profile associated with xGvHD lesions. In this context, we demonstrated robust accumulation of macrophages with M2-like polarization, marked by expression of arginase-1, accounting for a median of 8% (interquartile range = 2%–19%) of cells across all (human and mouse combined) infiltrating inflammatory/immune cells (Fig. 5d and Supplemental Figure 2). In contrast, macrophages with M1-like polarization, marked by expression of inducible nitric oxide synthase (iNOS), were invariably absent from all the examined xGvHD lesions (Fig. 5e). As observed with other markers, while some degree of arginase-1 variability was detected across treatment groups, the small sample sizes selected for the IHC study did not allow for any meaningful statistical comparisons (Supplemental Figure S2). In addition, regardless of the treatment-group distributions, no statistically significant correlation could be traced between lesion severity scores and the proportion of arginase-1-positive cells.

## Discussion

This work provides a detailed histological and immunohistochemical assessment of xGvHD lesions developed in NSG mice injected with human CAR T cells. xGvHD has been extensively described in diverse experimental contexts involving mouse humanization, from patient-derived tumor xenograft mouse models to mice with humanized immune systems.^11,28,32,39,43^ However, the murine xGvHD phenotype resulting from human CAR T cell administration has not been thoroughly characterized, despite this unintended disorder being a notorious cause of premature morbidity and mortality in treated animals.^8^ Our observations, extrapolated from a relatively large experimental cohort of NSG mice administered CAR T cells, suggest that salivary and lacrimal glands, lungs, skin, and external genitalia represent the most consistently and highly affected tissues during CAR T cell-driven xGvHD. This lesion distribution, together with the spectrum of the pathological changes described in our cohort of CAR T cell-treated NSG mice, is very similar to previous reports of xGvHD developed in other experimental contexts, including patient-derived tumor xenograft mouse models and mice transplanted with human peripheral blood mononuclear cells.^32,43,45^

Our evaluation revealed important variations in xGvHD phenotype progression across the examined experimental groups. In this context, the most significant observation was the robust development of xGvHD in CAR T cell-treated mice, but only minimal lesions or no changes in mice treated with NT T cells. This finding is not surprising considering that, unlike CAR T cells, NT T cells lack the same capacity for sustained expansion and persistent activation in response to tumor cells.^5^ Furthermore, we identified a clear trend toward increased xGvHD lesion severity in mice receiving CAR T cells with costimulatory domain A compared with those receiving CAR T cells with costimulatory domain B. This outcome highlights how different signaling pathways, resulting from diverse CAR constructs and associated costimulatory domains, can impact T cell activation and expansion, especially after engaging with the specific tumor target, affecting the initiation or aggravation of GvHD.^14,42^ This is also a well-known manifestation in patients treated with allogeneic CAR T cells, where the risk of developing an alloreactive response is greatly impacted not only by the clinical context but also by the characteristics of the CAR and the choice of costimulatory domains, which dictate T cell activation, persistence, and alloreactivity.^1,12,30,42^ However, a major limitation of our study is the inability to disclose and discuss the specific CAR constructs (including costimulatory domains), which precludes further speculation about possible underlying mechanisms, as well as the preclinical relevance and translational potential of the reported outcomes.

The observed inverse correlation between the severity of xGvHD and the degree of peritoneal carcinomatosis is not unexpected, given the growing body of clinical evidence suggesting that higher efficacy of CAR T therapy often comes at the cost of increased off-target toxicity, including GvHD.^26^ Nevertheless, our histological assessment of tumor burden has to be interpreted with caution because of the limitations of the sectioning approach, which does not allow for comprehensive screening of implants throughout the peritoneal cavity. In this context, more rigorous experimental approaches, such as in vivo or ex vivo imaging using tumor-specific tracers or reporters, would have provided a more precise quantification of carcinomatosis levels.

One of the primary objectives of our study was to characterize the murine myeloid cells involved in xGvHD lesions. Although our analysis was based on a relatively small sample size, it provided valuable insights into the critical interplay between human T cells and the mouse histiocytic population in shaping the xGvHD phenotype. In this context, we demonstrated that approximately 45% of the infiltrating mononuclear inflammatory/immune cells were murine in origin, with a prevalence of IBA1+ histiocytes, including relatively large numbers of CD86+ APCs and relatively small but constant fractions of CD11c+ dendritic cells and CD40+ APCs. Both CD86 and CD40 are important costimulatory molecules expressed on APCs, but they play distinct roles in the immune response and operate through different signaling pathways. While CD40 is critical for B cell-intrinsic processes, such as class switching and germinal center formation, CD86 is primarily involved in T cell activation.^15,25^ In this scenario, the high prevalence of CD86+ APCs associated with xGvHD likely reflects active interspecies immune cross-talk, specifically between human T cells and mouse APCs. Consistent with findings from other models of xGvHD, these results support the broader concept that the interaction between CD86 on murine APCs and CD28 on human CAR T cells is a key driver of xenoreactive T cell responses, playing a pivotal role in both initiating and amplifying immune activation across species barriers.^10,41^ Experimental and clinical evidence further emphasizes the importance of CD86 costimulation in promoting sustained T cell activation during GvHD.^4,19,23,24^ Taken together, these data highlight the potential therapeutic value of CD86 costimulation blockade for the prevention of GvHD.^22^ Nonetheless, as we failed to identify a statistically significant correlation between xGvHD lesion severity scores and percentage of CD86+ cells, further studies are needed to establish whether our preclinical observations can guide the strategic use of T cell costimulation blockade as a means to mitigate allogeneic CAR T cell-mediated GvHD.

In our study, the development of human CAR T cell-mediated xGvHD was accompanied by a striking shift toward M2-like polarization in murine macrophages, as demonstrated by robust arginase-1 expression and a complete lack of iNOS. In the clinical setting, variations in macrophage polarization play a significant role in shaping the severity and progression of GvHD.^16,33^ In general terms, M1 macrophage polarization prevails during the acute phase of GvHD, contributing to tissue damage and exacerbating the severity of the clinical signs through the secretion of potent proinflammatory cytokines (eg, interleukin [IL]-6, IL-1β, and tumor necrosis factor-alpha [TNF-α]).^46,47^ Conversely, macrophages tend to polarize toward the M2 phenotype during the chronic phase of GvHD. While this shift is associated with tissue repair and immune regulation, excessive activation and tissue accumulation of M2 macrophages carries the risk of fibrosis and organ dysfunction.^16^ The impact of this double-edged phenomenon of M2-like macrophage polarization on disease progression in human CAR T cell-mediated xGvHD in mice remains unclear. The predominance of M2-like polarization observed in our experiments may indicate the transition toward a chronic phase of xGvHD. In this context, the xenoreactive response mediated by human CAR T cells in immunocompromised mouse recipients may provide a valuable platform for testing therapeutic strategies designed to redirect M2 macrophage polarization and prevent irreversible chronic tissue damage.^7,48^ Interestingly, xenogeneic T cells were shown to produce the cytokines IL-10 and IL-4, which polarize macrophages toward an M2-like phenotype.^9^ Nonetheless, it should be noted that although the distinctions between acute and chronic GvHD are well established in human clinical practice, based largely on onset timing and characteristic clinical manifestations, these definitions are not readily applicable to describing the clinicopathological evolution of xGvHD in murine models.^31,38^ Therefore, any exploration of potential parallels between clinical and experimental GvHD must first acknowledge the critical pathophysiological distinctions between humans and mice. Recognizing these differences is crucial for accurately interpreting preclinical data and ensuring effective translation into human therapies. It is also important to note that while our work suggests a shift toward an M2-like phenotype at the time of analysis, macrophage activation and polarization are complex and dynamic states, and further analysis of these populations and their effects on tissue damage and repair is required.^34^

In conclusion, the present work demonstrates the critical contribution of murine histiocytes in the pathogenesis of human CAR T cell-mediated xGvHD. Our findings suggest that the murine APCs provide a costimulatory signal via CD86 during the xenoreactive human CAR T cell attack. Furthermore, this process is characterized by a striking shift toward M2-like polarization in murine macrophages, which pathogenetically might be linked to a progression toward a pro-fibrotic resolution of the tissue damage caused by the xenoreactive human CAR T cells.

## Supplemental Material

sj-pdf-1-vet-10.1177_03009858251391388 – Supplemental material for Characterization of host immune cell infiltrate in human CAR T cell-mediated xenogeneic graft versus host disease in NSG mice Supplemental material, sj-pdf-1-vet-10.1177_03009858251391388 for Characterization of host immune cell infiltrate in human CAR T cell-mediated xenogeneic graft versus host disease in NSG mice by Elinor Willis, Esha Banerjee, Jillian Verrelle, Arin Cox, Charles-Antoine Assenmacher and Enrico Radaelli in Veterinary Pathology
