# Supplementary material for: Characterization of host immune cell infiltrate in human CAR T cell-mediated xenogeneic graft versus host disease in NSG mice
Source: Vet Pathol. 2025 Nov 19;63(3):490–9. doi: 10.1177/03009858251391388 (PMC13036270; doi:10.1177/03009858251391388)
Supplement: sj-pdf-1-vet-10.1177_03009858251391388 – Supplemental material for Characterization of host immune cell infiltrate in human CAR T cell-mediated xenogeneic graft versus host disease in NSG mice [file sj-pdf-1-vet-10.1177_03009858251391388.pdf]

Supplemental Materials

Characterization of host immune cell infiltrate in human CAR T cell-mediated xenogeneic graft versus host disease in NSG mice.

Elinor Willis, Esha Banerjee, Jillian Verrelle, Arin Cox, Charles-Antoine Assenmacher, Enrico Radaelli

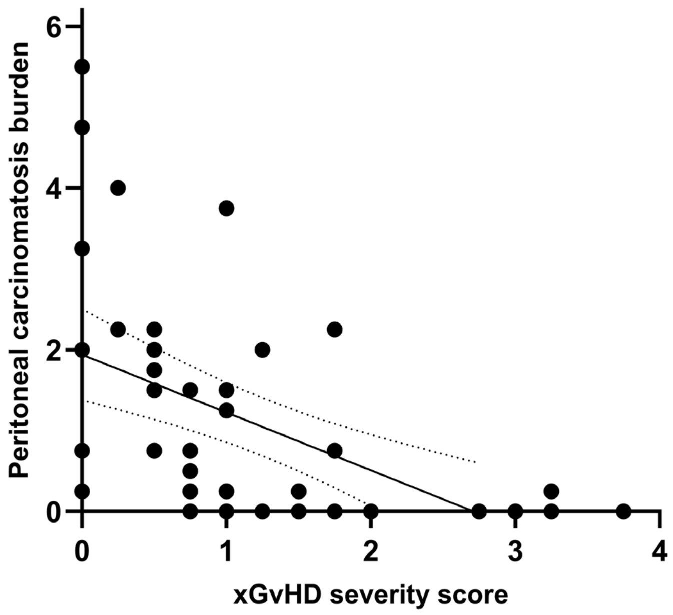

Supplemental Figure S1. Peritoneal carcinomatosis burden is negatively correlated with xenogeneic graft versus host disease (xGvHD) lesion severity. Peritoneal carcinomatosis in each animal was assigned a value according to the number and size of neoplastic implants. Spearman's correlation analysis revealed a strong negative relationship (r_s -0.6682; p<0.0001) between xGvHD lesion severity and peritoneal tumor burden. Each point represents one animal; some points overlap.

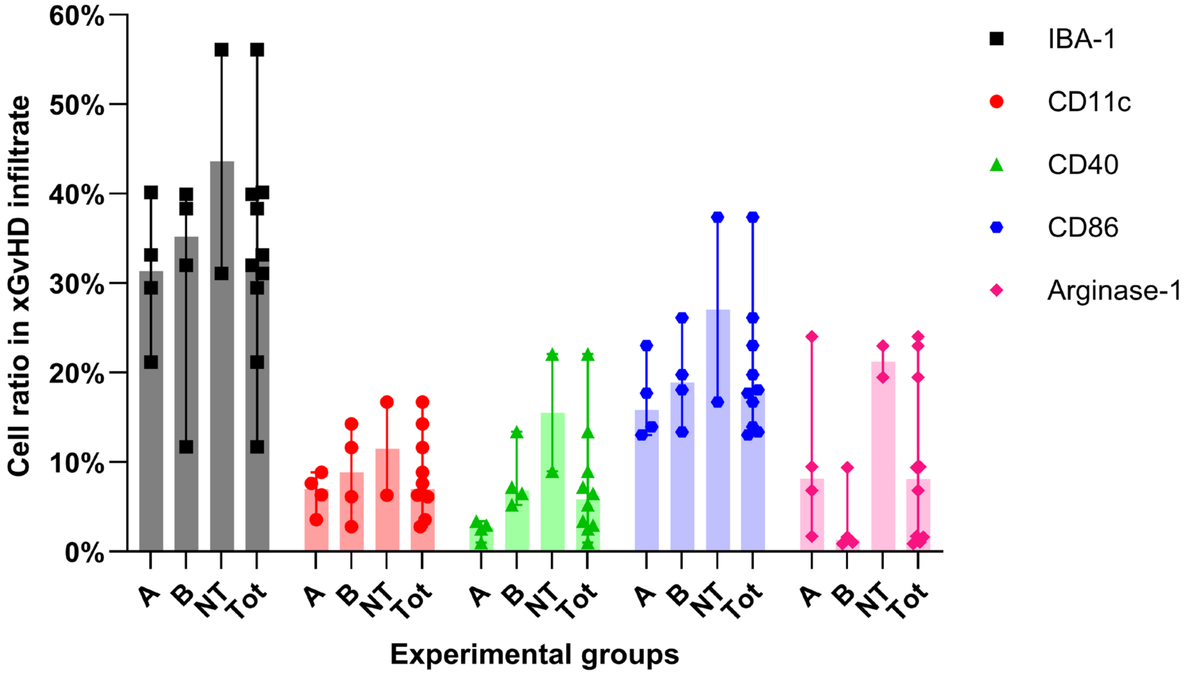

Supplemental Figure S2. Percent of murine histiocytes expressing markers of activation/antigen-presentation and macrophage polarization in xenogeneic graft versus host disease lesions by experimental group. Infiltrating cells were immunolabeled for each marker and positive cells were quantified by digital image analysis. Values were normalized to mouse-specific CD45. Each point is one animal; columns represent the median values. A, group treated with chimeric antigen receptor (CAR) T cells transduced with a construct containing costimulatory domain A; B, group treated with CAR T cells transduced with a construct containing costimulatory domain B; NT, nontransduced; Tot, total.

Supplemental Table S1. Criteria for semiquantitative scoring of xGvHD lesions.

Score	Tissue involvement by mononuclear cell infiltrate	Tissue damage associated with the mononuclear cell infiltrate
0 (unremarkable)	No tissue involvement	No tissue damage
1 (minimal)	< 10% of tissue	Very rare individual epithelial cell death in affected parenchymal organs, skin, and mucous membranes, but no fibrosis or evidence of tissue atrophy/loss or ulceration.
2 (mild)	10-25% of tissue	Multifocal individual epithelial cell death in affected parenchymal organs, skin, and mucous membranes, with or without modest fibrosis, but no evidence of tissue atrophy/loss or ulceration.
3 (moderate)	25-50% of tissue	Frequent individual to confluent epithelial cell death in affected parenchymal organs, skin, and mucous membranes, with modest fibrosis and limited tissue atrophy/loss or ulceration.
4 (severe)	> 50% of tissue	Extensive epithelial cell disruption in affected parenchymal organs, skin, and mucous membranes, with modest to marked fibrosis and severe tissue atrophy/loss or ulceration.

Supplemental Table S2. Details concerning primary antibodies and procedures used for chromogenic immunohistochemistry.

Antigen/ Target	Primary antibody	Source	Antigen retrieval	Working dilution	Incubation time	Positive (+) and/or negative (-) controls	Labeling platform and detection system
IBA1	Rb pAb #019-19741	WAKO	EDTA based pH 6.0 solution (#AR9961), 20 minutes, 98 °C	1:1500	45 minutes RT	(+) Spleen from an adult naïve C57BL/6J mouse	Leica BOND RXm, Bond Polymer Refine Detection Kit (#DS9800)
Arginase-1	Rb mAb #93668	Cell Signaling Technology	EDTA based pH 9.0 solution (#AR9640), 20 minutes, 98 °C	1:300	45 minutes RT	(+) Brain from a C57BL/6J mouse infected with *T. gondii*<br>(-) Human tonsil	Leica BOND RXm, Bond Polymer Refine Detection Kit (#DS9800)
CD11c (mouse specific)	Rb mAb #97585	Cell Signaling Technology	EDTA based pH 9.0 solution (#AR9640), 20 minutes, 98 °C	1:150	45 minutes RT	(+) Spleen from an adult naïve C57BL/6J mouse<br>(-) Human tonsil	Leica BOND RXm, Bond Polymer Refine Detection Kit (#DS9800)
CD40 (mouse specific)	Rb mAb #86165	Cell Signaling Technology	EDTA based pH 9.0 solution (#AR9640), 20 minutes, 98 °C	1:150	45 minutes RT	(+) Spleen from an adult naïve C57BL/6J mouse	Leica BOND RXm, Bond Polymer Refine Detection Kit (#DS9800)
CD45 LCA (human specific)	Rb mAb #13917	Cell Signaling Technology	EDTA based pH 9.0 solution (#AR9640), 20 minutes, 98 °C	1:300	45 minutes RT	(+) Human tonsil<br>(-) Lymphoid tissues from an adult Crl:CD1(ICR) mouse	Leica BOND RXm, Bond Polymer Refine Detection Kit (#DS9800)
CD45 LCA (mouse specific)	Rb mAb #70257	Cell Signaling Technology	EDTA based pH 9.0 solution (#AR9640), 20 minutes, 98 °C	1:300	45 minutes RT	(+) Lymphoid tissues from an adult Crl:CD1(ICR) mouse<br>(-) Human tonsil	Leica BOND RXm, Bond Polymer Refine Detection Kit (#DS9800)
CD86 (mouse specific)	Rb mAb #19589	Cell Signaling Technology	EDTA based pH 9.0 solution (#AR9640), 20 minutes, 98 °C	1:300	45 minutes RT	(+) Spleen from an adult naïve C57BL/6J mouse	Leica BOND RXm, Bond Polymer Refine Detection Kit (#DS9800)
iNOS (mouse specific)	Rb pAb #ab15323	Abcam	EDTA based pH 9.0 solution (#AR9640), 20 minutes, 98 °C	1:100	45 minutes RT	(+) Brain from a C57BL/6J mouse infected with *T. gondii*<br>(-) Human tonsil	Leica BOND RXm, Bond Polymer Refine Detection Kit (#DS9800)

Abbreviations used in the table: Rb, rabbit; mAb, monoclonal antibody; pAb, polyclonal antibody; RT, room temperature.
